# Multi-center evaluation of machine learning-based radiomic model in predicting disease free survival and adjuvant chemotherapy benefit in stage II colorectal cancer patients

**DOI:** 10.1186/s40644-023-00588-1

**Published:** 2023-08-03

**Authors:** Hui Zhu, Muni Hu, Yanru Ma, Xun Yao, Xiaozhu Lin, Menglei Li, Yue Li, Zhiyuan Wu, Debing Shi, Tong Tong, Haoyan Chen

**Affiliations:** 1 Department of Diagnostic Radiology, Department of Oncology, Fudan University Shanghai Cancer Center, Shanghai Medical College, Fudan University, 270 DongAn Road, Shanghai, 200032 China; 2 State Key Laboratory for Oncogenes and Related Genes, Key Laboratory of Gastroenterology and Hepatology, Ministry of Health, Division of Gastroenterology and Hepatology, Renji Hospital, School of Medicine, Shanghai Jiao Tong University, Shanghai Cancer Institute, Shanghai Institute of Digestive Disease, Shanghai, China; 3 Department of Radiology, Peking University People’s Hospital, 11 Xizhimen South St, Beijing, 100044 China; 4 Department of Radiology, Rui Jin Hospital, Shanghai Jiao Tong University School of Medicine, No. 197, Rui Jin Er Rd, Shanghai, 200025 China; 5 Department of Colorectal Surgery, Department of Oncology, Fudan University Shanghai Cancer Center; Shanghai Medical College, Fudan University, 270 DongAn Road, Shanghai, 200032 China

**Keywords:** Radiomics, Computed tomography, Prognosis, Stage II colorectal cancer, Adjuvant chemotherapy

## Abstract

**Background:**

Our study aimed to explore the potential of radiomics features derived from CT images in predicting the prognosis and response to adjuvant chemotherapy (ACT) in patients with Stage II colorectal cancer (CRC).

**Methods:**

A total of 478 patients with confirmed stage II CRC, with 313 from Shanghai (Training set) and 165 from Beijing (Validation set) were enrolled. Optimized features were selected using GridSearchCV and Iterative Feature Elimination (IFE) algorithm. Subsequently, we developed an ensemble random forest classifier to predict the probability of disease relapse.We evaluated the performance of the model using the concordance index (C-index), precision-recall curves, and area under the precision-recall curves (AUC_PR_).

**Results:**

A radiomic model (namely the RF5 model) consisting of four radiomics features and T stage were developed. The RF5 model performed better than simple radiomics features or T stage alone, with higher C-index and AUC_PR_, as well as better sensitivity and specificity (C-index_RF5_: 0.836; AUC_PR_ = 0.711; Sensitivity = 0.610; Specificity = 0.935). We identified an optimal cutoff value of 0.1215 to split patients into high- or low-score subgroups, with those in the low-score group having better disease-free survival (DFS) (Training Set: *P* = 1.4e-11; Validation Set: *P* = 0.015). Furthermore, patients in the high-score group who received ACT had better DFS compared to those who did not receive ACT (*P* = 0.04). However, no statistical difference was found in low-score patients (*P* = 0.17).

**Conclusion:**

The radiomic model can serve as a reliable tool for assessing prognosis and identifying the optimal candidates for ACT in Stage II CRC patients.

**Trial registration:**

Retrospectively registered.

**Supplementary Information:**

The online version contains supplementary material available at 10.1186/s40644-023-00588-1.

## Introduction

Radiomics is a mathematical technique used to analyze medical images in a quantitative manner, with the goal of providing clinicians with additional data to aid in diagnosis and treatment [1]. Imaging evaluations have traditionally been employed to monitor treatment response in tumors. However, with the advent of radiomics, it is now possible to extract and analyze thousands of image features to predict treatment response [2, 3]. The radiomics process involves acquiring and preprocessing vast volumes of medical image data, segmenting tumors to analyze specific regions, and mining the data for feature extraction and modeling [4]. Radiomics has enormous research potential in the age of personalized medicine, as it can improve survival prediction and help discover new molecular pathways in tumor development, rather than serving solely as a tool for clinical decision-making [5].

Colorectal cancer (CRC) is the third most common cause of cancer death in both men and women in the United States and ranks the second considering both genders together [6]. Among early stages of the disease, Stage II CRC (T3 N0M0 and T4N0M0) is defined in the 8th edition of the American Joint Committee on Cancer (AJCC) staging manual, indicating no lymph node or distant organ metastasis. It accounts for about one-third of patients who have undergone curative resection for CRC [6, 7].

Patients with Stage II CRC usually demonstrate widely heterogeneous prognosis with the 5-year overall survival (OS) varying from 50 to 80% [8]. A higher T stage in Stage II CRC patients was associated with higher risk for disease recurrence. Therapeutic strategy tailored for Stage II CRC remains a challenge since 'one size fits all' is not adequate for this setting [7, 9].

For patients with medium to high risk stage II colorectal cancer, adjuvant chemotherapy (ACT) is the established postoperative treatment [10, 11]. Regrettably, to date, none of the clinically validated biomarkers have been able to precisely determine which patients with Stage II CRC will derive benefits from ACT [12, 13]. Although CT features assessments have proven to be valuable in predicting the prognosis of colorectal cancer (CRC) patients, an effective method that integrates multiple imaging biomarkers into a predictive signature has not yet been devised [14–16]. Recent studies demonstrate that radiomics model can predict complete response and recurrence risk in a non-invasive manner [17, 18]. However, it remains evidence-deficient that the radiomics potentially exert effects in benefit evaluation of ACT for stage II CRC patients.

Herein, we hypothesized that a set of CT-derived radiomics, could act as predictive biomarkers to evaluate disease-free survival (DFS) for stage II CRC patients. A noninvasive CT-based radiomics signature was developed and validated to identify the optimal candidates who benefit most from postoperative ACT, thus offering practical clinical value.

## Material and methods

### Study design and patients

Records from September 2012 to June 2019 of the colorectal surgery, radiology and pathology departments of Fudan University Shanghai Cancer Center (Shanghai, China) (Training set), and Peking University People’s Hospital (Beijing, China) (Validation set) were reviewed and cross-referenced. To create a study group of suitable cases, we used the following inclusion criteria: (a) pathologically confirmed stage II CRC, (b) available clinical data (including TNM, survival information, age, sex), (c) available contrast-enhanced CT (portal venous phase) images. We chose portal venous phase images considering the operability of image segmentation. Exclusion criteria were as below: (a) patients received any treatments (radiotherapy, chemotherapy, or chemoradiotherapy) before CT scan, (b) clinical data was incomplete, (c) insufficient image quality. A total of 478 stage II CRC patients were enrolled, of which 313 patients from Shanghai was trained for the model building and the else from Beijing was considered as external validation. It was defined that disease-free survival (DFS) was the time from surgery until either disease progression or death from any cause. We adopted a duration of five-year DFS as the standard to define patients’ disease status. The disease relapse was classified as patients who experienced disease progression or death from any cause within 5 years; those with prolonged survival or disease release were considered as the non-relapse. Tumor staging was performed referring to the American Joint Committee on Cancer TNM Staging Manual, eighth Edition [6]. Patients with ACT mainly adopted oxaliplatin plus 5-fluorouracil, in addition to Fluorouracil monotherapy strategies. This retrospective study was approved by our institutional review board, and informed consent was waived.

### CT examination and image segmentation

All patients underwent enhanced CT examination before surgery. The details of the CT protocol were shown in Supplementary methods. Portal venous phase CT images were loaded into ITK-SNAP version 3.6.0 (an open-source image analytics software, www.itksnap.org) for tumor segmentation. For each tumor, an experienced abdominal radiologist (reader 1) reviewed all axial slices and selected one single slice with the largest tumor area. A two-dimensional region of interest (ROI) was manually delineated along the outline of the tumor. The task of verifying the segmentation profiling carried out by the first radiologist fell under the purview of the second radiologist (reader 2). In case of any discrepancies, they collaborated and settled the differences through discussion.

### Development and validation of the radiomics-based model

Radiomics features were extracted from the ROI of each CRC samples, including features of first order statistics(*N* = 161), features of shape(*N* = 14), features of grey-level co-occurrence matrix (GLCM) (*N* = 198), features of grey-level run-length matrix (GLRLM)(*N* = 144), features of grey-level size-zone matrix (GLSZM) (*N* = 144), features of gray-level dependence matrix (GLDM) (*N* = 126) using PyRadiomics on Python (version 3.7.10). All the features were analyzed with the PyRadiomics package and the procedure of feature extraction was performed using the default setting.The discriminative radiomic features were identified using a two-sided blocked Wilcoxon rank- sum test implemented in the R. Following the discriminative radiomic features, we built random forest models in the scikit-learn (V.0.19.2) package with stratified tenfold cross validation to distinguish the CRC with recurrence or not. The training group (*N* = 313), for which we developed the model for prediction, and the test group (*N* = 165), on which we evaluated the trained classifier. The features used for model building consist of discriminative radiomics features as well as the patient metadata features including age, sex, and T stage. The RF models were built with 501 estimator trees and each tree had 10% of the total features. A total of 10,000 random forest classifier models were evaluated with different combinations of hyperparameters: max_features = “auto”; n_estimators ranging from 100 to 1,000 with an interval of 100; max_depth ranging from 2 to 20 with an interval of 2; min_samples_leaf ranging from 2 to 20 with an interval of 2; and min_ samples_split ranging from 2 to 20 with an interval of 2. Then an IFE (Iterative Feature Elimination) step was used to optimize the performance of subsequent RF models. The top features from the top-performing model were selected as “best features”. To build a random forest classifier with the best hyperparameters, we implemented the exhaustive grid search approach using the GridSearchCV function to the training dataset with ten-fold cross-validation. The permutation-based importance (function PermutationImportance) from the ELI5 Python package (https://eli5.readthedocs.io/) was finally utilized to compute the feature importance for models. Using the pROC package, the Youden's index method was utilized to determine the ideal thresholds for the radiomics-based score that would effectively differentiate between relapse and non-relapse cases in the training set. The Chi-square test was utilized to evaluate the discriminatory ability on the model using the optimal thresholds of the radiomics-based score. The concordance index (C-index), precision-recall curves and area under the precision-recall curves (AUC_PR_) were calculated for radiomics-based model performance evaluation. Model predictive performance was measured by multi-metrics including sensitivity, specificity, accuracy, positive predictive value (PPV) and negative predictive value (NPV). Kaplan–Meier survival analysis was done and compared between high-score and low-score groups in the training set and validation set.

### Statistical analysis

For continuous variables, we used the actual values of each of the variables to generate analysis; for categorical variables, such as sex (male or female), T stage (T3 or T4), ACT status (with or without ACT) and radiomics-based score group (high or low), binary values were used. To compare the distributions of relapse probability generated by the radiomics-based model among different cohorts, the two-sided Mann–Whitney U test was used. We calculated the C-index of our model predictions with the survcomp package. We visualized ROC and calculated the AUC using the pROC package. The Kaplan–Meier plot, log-rank P values and hazard ratios (HR) were generated by the survminer package. Survival curves were generated in accordance with the Kaplan–Meier method and compared by the log-rank test. We used the t-test for continuous variables comparison and the Chi-square test for categorical variables, as appropriate. The coefficients were applied to the construction of the radiomics-based model. All the aforementioned statistical analysis was implemented with R software (version 4.0.1). A two-sided *P* value < 0.05 was considered significant.

## Results

### Baseline characteristics of the patients

In this study, we investigated CT image data from two clinical centers (Shanghai and Beijing) to establish a radiomics-based model for prognosis evaluation. The demographic characteristics were presented in Table 1. Of the total 313 patients from Shanghai, serving as training set, were enrolled in this study, 187 (59.7%) were men, and the mean age was 59.20 years. The cohort of 165 qualified patients from Beijing were included as the external validation group. Besides, patients (*N* = 313) from the training set provided the information on ACT, in which 216(69.0%) patients had received ACT.

**Table 1:** Overview on baseline characteristics of the patients in the study

Characteristic	Training Set (*N* = 313)	Validation Set (*N* = 165)
**Gender (%)**		
Male	187(59.7)	79(47.9)
Female	126(40.3)	86(52.1)
**Age, years, mean ± SD**	59.20 ± 12.14	66.54 ± 12.90
**DFS,months, mean ± SD** **DFS status**	44.96 ± 17.12	59.4 ± 26.02
Relapse	46(14.7)	20(12.1)
Non-relapse	267(85.3)	145(87.9)
**Adjuvant chemotherapy (%)**		
Without ACT	97(31.0)	–
With ACT	216(69.0)	–
**Radiomics score, mean ± SD**	0.1464 ± 0.0843	0.1535 ± 0.0824

Baseline characteristics of the patients

### Development and validation of the machine learning-based radiomic model

**Fig. 1:**
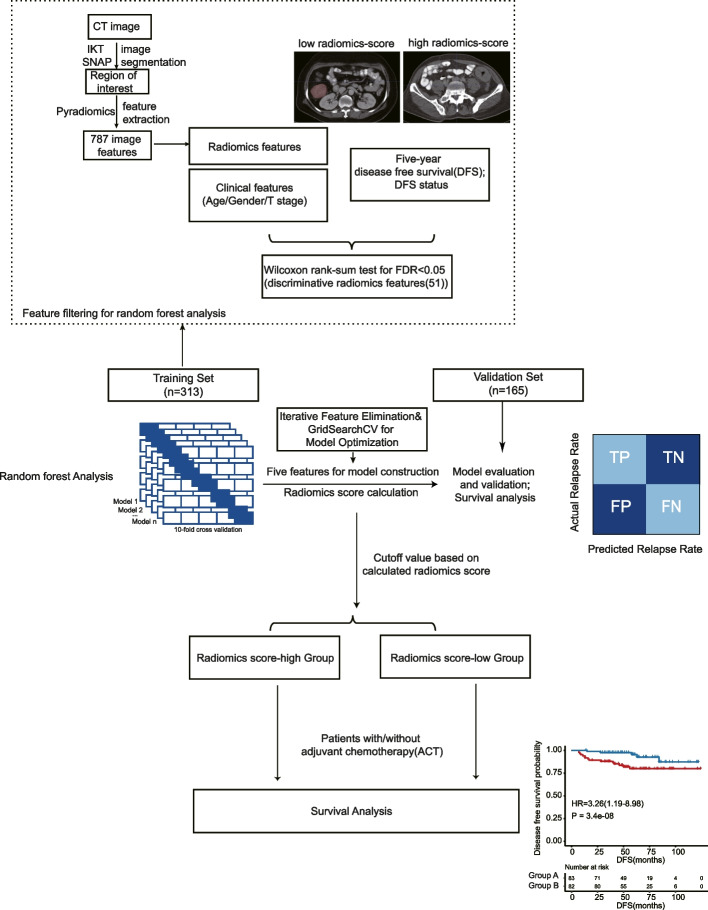
The overview of CT image radiomics workflow and study flowchart. Two cohorts, including the training set (313) and the validation set (165) were collected and analyzed using the random forest model training and testing procedure

To calculate the probability of disease relapse, we developed an ensemble learning random forest classifier. The CT image radiomics workflow and study flowchart was shown in Fig. 1. In total, 787 radiomics features were extracted from each patient’s CT image. Wilcoxon rank-sum test revealed 51 discriminative radiomics features with the FDR less than 0.05. Following the application of IFE, a particular model was chosen based on the following hyperparameters: n_estimators = 1,000, max_depth = 8, min_samples_leaf = 20, and min_samples_split = 2. This model demonstrated the highest average accuracy of 0.7559.

**Fig. 2:**
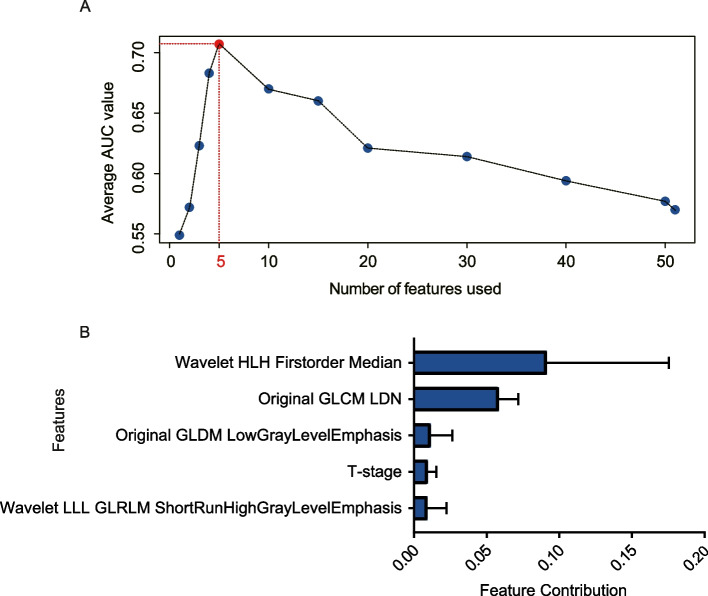
Radiomics feature filtering and selection criterion for model construction. **A** The optimal feature number for model construction using the AUC value. **B** Feature contribution of the 5 model features calculated in the training set to predict DFS. The error bars denote standard deviation of feature contribution

The study identified five features, namely wavelet-HLH (wavelet HLH firstorder Median), GLDM-IDN(original GLDM InverseDifferenceNormalized (IDN)), GLDN- LowGrayLevelEmphasis(original gldm LowGrayLevelEmphasis), T stage, and wavelet-LLL-GLRLM(wavelet LLL GLRLM ShortRunHighGrayLevelEmphasis), that demonstrated the highest performance in predicting relapse or non-relapse in stage II CRC patients (Fig. 2A). These features were used to develop a predictive model using binary classification and tenfold cross-validation on the training data. The model was able to aggregate the predictive effects of selected clinical and radiomics features to derive a specific probability of tumor relapse. Based on the individual levels of these five predictors, a radiomics-based score was generated for each patient (Fig. 2B). In addition, a RF4 model was also developed using the same method, but with only four of the filtered radiomics features (wavelet-HLH (wavelet HLH firstorder Median), GLDM-IDN(original GLDM InverseDifferenceNormalized (IDN)), GLDN- LowGrayLevelEmphasis(original gldm LowGrayLevelEmphasis).

### Machine learning-based radiomic model performance assessment

**Fig. 3:**
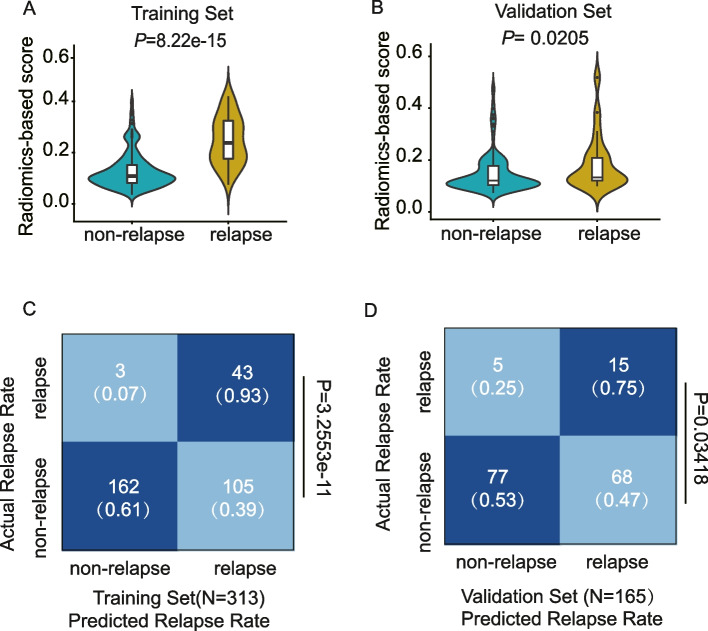
The performance on radiomics score derived from prediction model across different cohorts. **A**, **B** Comparison of response probability distributions calculated by RF5 model between non-relapse and relapse groups, in the training set and validation set, respectively. Two-sided *P* values were calculated using the wilcoxon rank-sum test. **C**, **D** Confusion matrices revealed predicted outcomes generated by RF5 model, as indicated, in training set and validation set, respectively. Statistical analysis was conducted based on the predictive value and actual value of relapse and non-relapse using the cut off value of radiomics score defined in the training cohort, Chi-square test

**Fig. 4:**
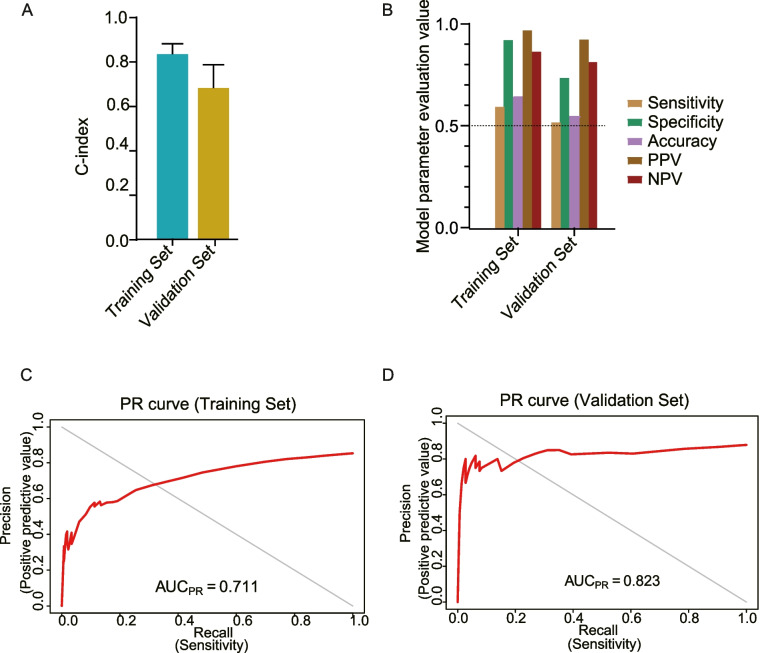
Radiomics-based Model performance assessments. **A** Performance measurements of RF5 model illustrated by sensitivity, specificity, accuracy, positive predictive value (PPV) and negative predictive value (NPV). **B** Comparison of concordance index (C-index) a for predicting disease-free survival (DFS) in the training and validation sets. **C**, **D** Precision-recall curve (PRC) and Prevalence curve assessed for statistical modeling in training set and validation set, respectively

The study found that the radiomics-based score, which is a continuous probability calculated by the random forest model using the selected five features, was significantly associated with relapse status in both the training and validation sets (Fig. 3A, B). The optimal cut-point on the radiomics-based score had excellent predictive value, as demonstrated by the confusion matrix (Fig. 3C, D). The performance of the model was further evaluated and compared using the C-index(C-index_training_ = 0.836; C-index_validatio*n*=_0.682) (Fig. 4A), and it was found that the RF5 model had significantly higher predictive performance than the RF4 model and the T stage (C-index_RF5_ = 0.836; C-index_RF4_ = 0.729; C-index_T stage_ = 0.614) (Supplementary Fig. 1A, B). The RF5 model was found to have fairly strong and consistent performance in predicting outcomes, as evaluated by metrics such as sensitivity, specificity, accuracy, PPV, and NPV(Training set: Sensitivity = 0.610,Specifity = 0.935, Accuracy = 0.655, PPV = 0.982, NPV = 0.877; Validation set: Sensitivity = 0.531,Specifity = 0.75, Accuracy = 0.558, PPV = 0.939, NPV = 0.827) (Fig. 4B). The precision-recall curve was used to assess the statistical modeling, and the RF5 model was found to have superior performance, as indicated by the AUC_PR_ in predicting relapse and non-relapse across different cohorts(Training set: AUC_PR_ = 0.711; Validation set: AUC_PR_ = 0.823) (Fig. 4C, D).

### Prognostic prediction performance of machine learning-based radiomic model for act

**Fig. 5:**
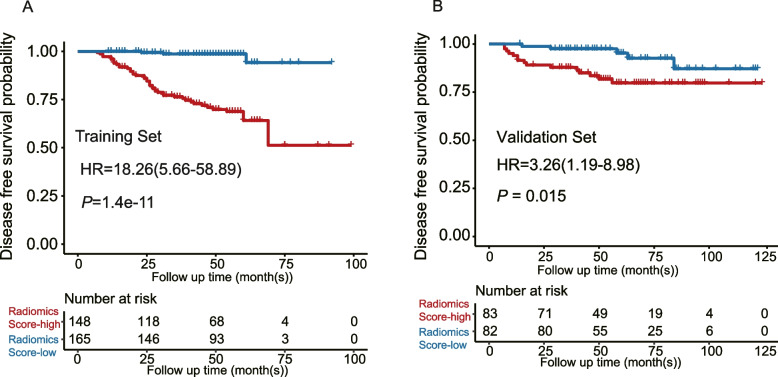
The prognosis value of predicting DFS in the (**A**). training set(*N* = 313) and (**B**). validation set(*N* = 165) respectively. Two-sided *P* values for comparison of DFS were computed using the log-rank test

To determine the optimal cut-off for the radiomics-based score, we utilized the ROC analysis within the R package "survivalROC". The optimal cutoff value was identified to be 0.1215 in the training set, which was subsequently used to divide all cohorts into score-high and score-low groups. We found that the score-low group was significantly associated with longer DFS compared to patients classified as the score-high group in all sets(Training set: *P* = 1.4e-11, HR = 18.26, 95% confidence interval (CI) = 5.66–58.89; Validation set: *P* = 0.015,HR = 3.26, 95% CI = 1.19–8.98) (Fig. 5A, B).

**Fig. 6:**
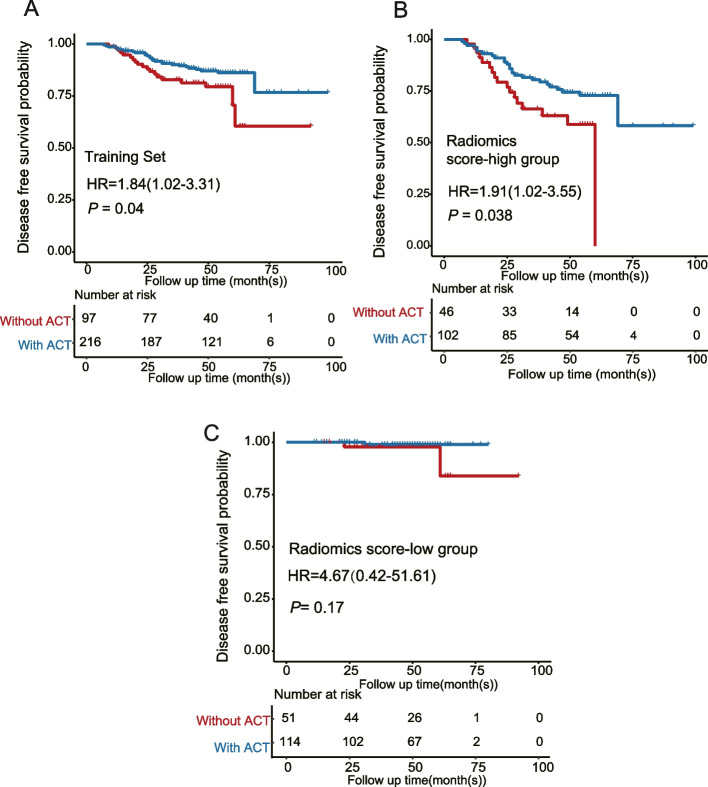
The prognosis value for predicting adjuvant chemotherapy (ACT) benefit in the training set using the cutoff value of radiomics score (optimal cutoff value of 0.1215). **A** Survival analysis of ACT benefit in the training set. **B** Survival analysis of ACT benefit in patients with low radiomics score. **C** Survival analysis of ACT benefit in patients with high radiomics score. Two-sided *P* values for comparison of DFS were computed using the log-rank test

Due to the relatively complete information on ACT in the training set, we performed survival analysis to distinguish stage II CRC patients who would benefit more from ACT in terms of DFS. Notably, Using Kaplan–Meier survival analysis, we found a significant difference in prognosis between stage II CRC patients who received ACT and those who did not. Specifically, patients who received ACT had a better DFS rate(HR = 1.84; 95%CI = 1.02–3.31; *P* = 0.04) (Fig. 6A). Interestingly, when stratifying patients based on the previously calculated cutoff value, patients who received ACT in the high-score group had a longer survival time compared to patients who did not receive ACT (HR = 1.91, 95%CI = 1.02–3.55, *P* = 0.038) (Fig. 6B). However, there was no significant difference in DFS between patients who received ACT and those who did not in the low-score group (HR = 4.67; 95%CI = 0.42–51.61; *P* = 0.17) (Fig. 6C). These findings indicated that patients in the high-score group who received ACT might benefit from it and should be recommended for treatment, whereas patients in the low-score group, who had a lower risk of relapse, might benefited less from ACT and should not be recommended for treatment.

## Discussion

We utilized a machine learning approach to analyze clinical data and accessible radiomic features from multi-center data originating from both the Shanghai cohort (training set) and the Beijing cohort (validation set) to identify key factors for evaluating the DFS of Stage II CRC patients. Pooling the data from different origins for statistical analysis might cause the potential heterogeneity and confounders. To address this issue, we performed tenfold cross validation and ensemble learning random forest classifier model with five features via the IFE and model optimization step to improve the robustness in the training set (Shanghai cohort), Subsequently, we applied the model to the validation set (Beijing cohort) to confirm its validity.

Our study also validates the substantial significance of the conventional TNM staging system in identifying a tumor's risk level, with a higher T stage indicating poorer clinical outcomes. Furthermore, our model, which incorporates both clinical and radiomics features, demonstrated superior predictive performance for DFS compared to models based solely on either T stage or radiomics features. Taken together, the results demonstrate that our radiomics-based model can accurately predict the DFS of Stage II CRC patients.

Furthermore, the results suggest that a radiomics-based model has the ability to predict the effectiveness of ACT in patients with stage II CRC. Presently, the decision to administer ACT is based on several clinical and pathological evaluations. Nonetheless, there are no dependable biomarkers available to determine patients who would benefit from longer ACT treatment. Recent studies have explored the use of non-invasive radiomic biomarkers to predict response to neoadjuvant chemoradiotherapy in rectal cancer [19, 20]. Our results showed that patients in the low-score group did not exhibit a significant difference in 5-year DFS between those who received postoperative ACT and those who did not, whereas patients in the high-score group did show a significant difference. Hence, patients in the low-score group do not require post-operative ACT as it may not be beneficial, and unnecessary treatment may lead to additional toxicity. Conversely, postoperative ACT is recommended for patients in the high-score group as those without it had an inferior 5-year DFS compared to those who received it. It is worth noting that radiomic signatures have recently been employed to predict outcomes and ACT benefits in lung adenocarcinoma and gastric cancer [21, 22]. Since the radiomic signature is based on the pretreatment CT image, it captures the tumor's biological properties that are independent of treatment. Therefore, we believe that the radiomic signature has the potential to become a useful clinical tool in the management of CRC patients.

Our study has some limitations. Firstly, we extracted 2D imaging features from a single slice instead of 3D imaging features from the entire tumor volume due to the ease of operation for radiologists. Secondly, complete ACT data was not available in the validation set, which made it challenging to assess external data for ACT benefit model evaluation. Moreover, potential limitations or biases may exist, especially in the context of any subjective evaluation such as inter-reader agreement, observer variability, or imaging feature capture. Standardized evaluation protocols and blinded assessments could reduce the bias and enhance outcome accuracy. To determine the validation group's appropriate sample size, we performed a power analysis in both the training and validation datasets. A power value of 0.8 or higher is typically considered adequate for sample size in this context. For this study, the estimated power value was 0.80, indicating that both the training and validation group sample sizes were adequate. In the future, a larger sample size and multi-center testing will be used to evaluate the proposed model.

## Conclusion

We developed and validated a noninvasive machine learning-based radiomic model for predicting relapse rate in stage II CRC patients referring to the DFS. This easy-to-use model can identify optimal candidates for postoperative ACT and ensure they may benefit from it. Although these findings need to be validated in large-scale prospective studies, we believe that our results have the potential to serve as a non-invasive alternative to personalized treatment in precision medicine.

### Supplementary Information

**Additional file 1:** **Supplementary Methods.** CT examinations.

**Additional file 2:** **Supplementary Figure 1.** (A). Comparison of concordance index (C-index) a for predicting disease-free survival (DFS) among RF5 model, RF4 model and T Stage in the training set (*n* = 313). (B). Comparison of C-index a for predicting DFS among RF5 model, RF4 model and T Stage in the validation set (*n *= 165).

## Data Availability

All data and codes were available from the corresponding author upon reasonable requests.

## Abbreviations

ACT: Adjuvant chemotherapy
AJCC: American Joint Committee on Cancer
AUC: Area under the curve
AUPRC: Precision-recall curves and area under the precision-recall curves
CRT: Chemotherapy and radiation therapy
CRC: Colorectal cancer
C-index: Concordance index
CT: Computed tomography
DFS: Disease-free survival
FPR: False positive rate
GLCM: Grey-level co-occurrence matrix
GLRLM: Grey-level run-length matrix
GLSZM: Grey-level size-zone matrix
GLDM: Gray-level dependence matrix
HR: Hazard ratio
IFE: Iterative Feature Elimination
NPV: Negative predictive value
PPV: Positive predictive value
ROC: Receiver operating characteristic
ROI: Region of interest
