# Supplementary material for: Multi-center evaluation of machine learning-based radiomic model in predicting disease free survival and adjuvant chemotherapy benefit in stage II colorectal cancer patients
Source: Cancer Imaging. 2023 Aug 3;23:74. doi: 10.1186/s40644-023-00588-1 (PMC10401876; doi:10.1186/s40644-023-00588-1)
Supplement: Supplementary file 1 — Additional file 1: Supplementary Methods. CT examinations. [file 40644_2023_588_MOESM1_ESM.docx]

Supplementary Methods: CT examinations

All enrolled patients underwent similar scan setup but with different various CT scanners, contrast agents and scanning parameters. All CT scanners were multidetector row CT, including Somatom Sensation 40 and Somatom Sensation 64, Siemens AG, Medical Solutions, Business Unit CT, Forchheim, Bavaria, Germany. All contrast agents were nonionic, including iopamidol, 300 mg iodine/mL, Iopamiro;

Bracco Sine, Shanghai, China or iohexol, 300 mg iodine/mL, Omnipaque 300; Amersham, Shanghai, China. For the contrast-enhanced scanning, each patient underwent imaging both in the arterial phase and in the portal venous phase (at the rate of 3 ml/s, with a 25 or 60 s delay, respectively). The contrast agents were administered by intravenous bolus injection into an antecubital vein by using power injectors (Ulrich Medizintechnik, Germany). The dose of the contrast agent was 1.5 ml/kg body weight. All patients were overnight fasted prior to CT scanning. The scanning parameters for the CT examinations were as follows: tube tension, 120 kV; tube current, 250-350 mA; slice thickness, 5 mm. The MDCT scanning was performed during inspiratory breath hold. The scanning range were from the diaphragmatic dome to the pubic symphysis, covering the entire colorectal region. The original images with 5-mm slice thickness were reconstructed into 1-2mm slice thickness and uploaded to the picture archiving and communication system (PACS, GE Healthcare-Centricity RIS CE V2.0; GE Medical Systems, Fairfield, Conn).
