# Supplementary figures and images for: Multi-center evaluation of machine learning-based radiomic model in predicting disease free survival and adjuvant chemotherapy benefit in stage II colorectal cancer patients
Source: Cancer Imaging. 2023 Aug 3;23:74. doi: 10.1186/s40644-023-00588-1 (PMC10401876; doi:10.1186/s40644-023-00588-1)

Supplementary Figure 1

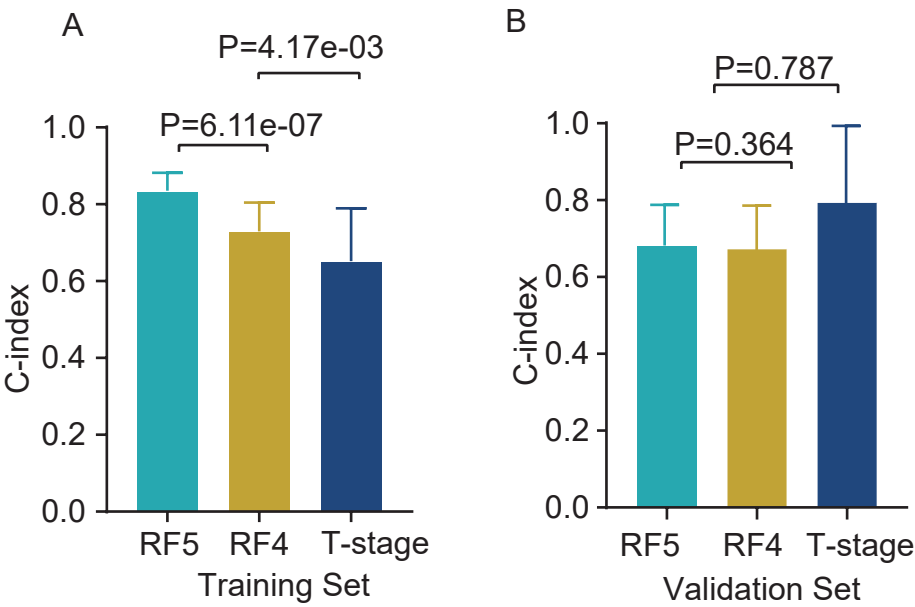

Supplement: Supplementary file 2 — Additional file 2: Supplementary Figure 1. (A). Comparison of concordance index (C-index) a for predicting disease-free survival (DFS) among RF5 model, RF4 model and T Stage in the training set (n = 313). (B). Comparison of C-index a for predicting DFS among RF5 model, RF4 model and T Stage in the validation set (n = 165). [file 40644_2023_588_MOESM2_ESM.pdf]
